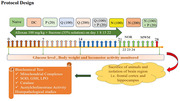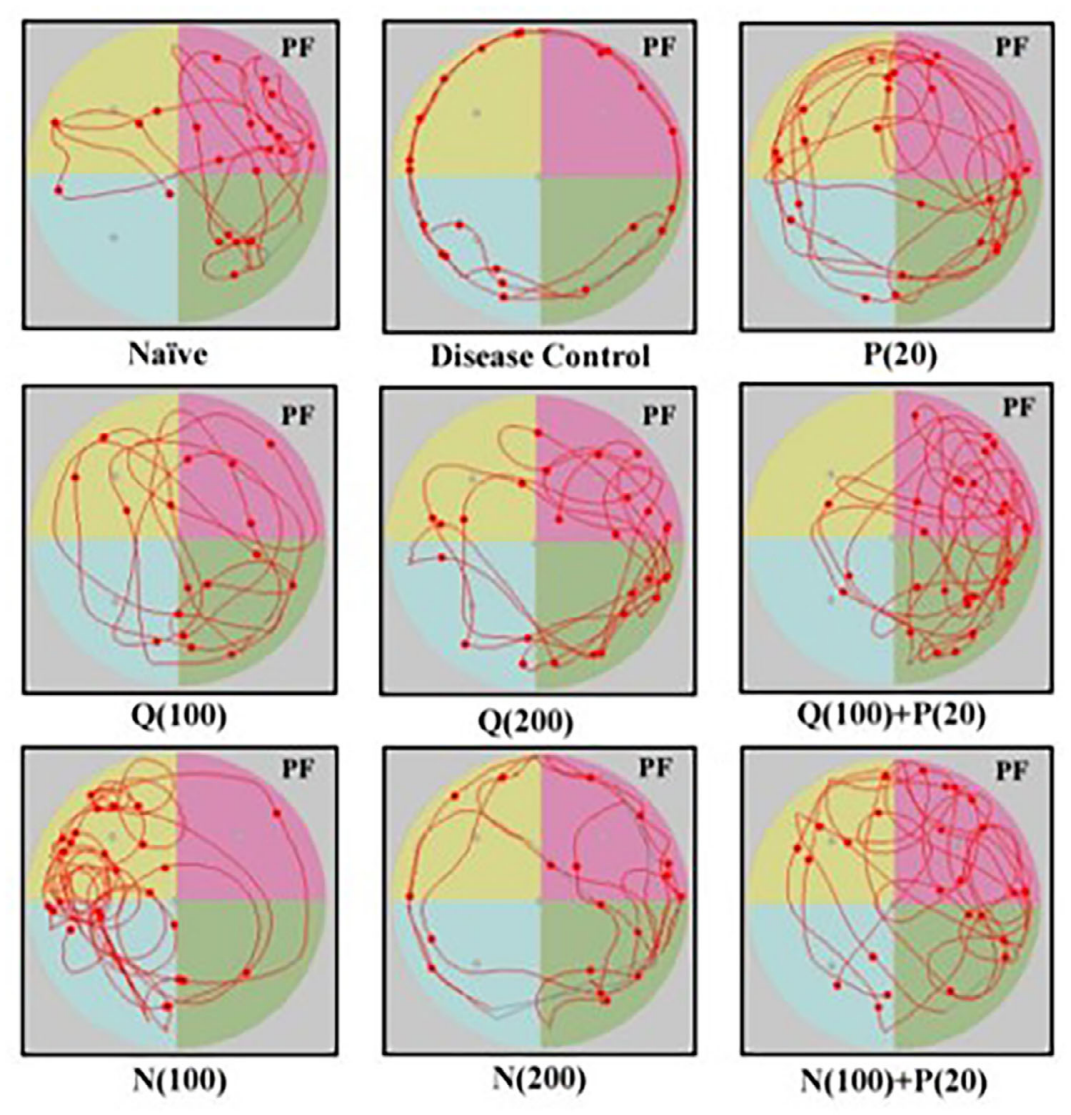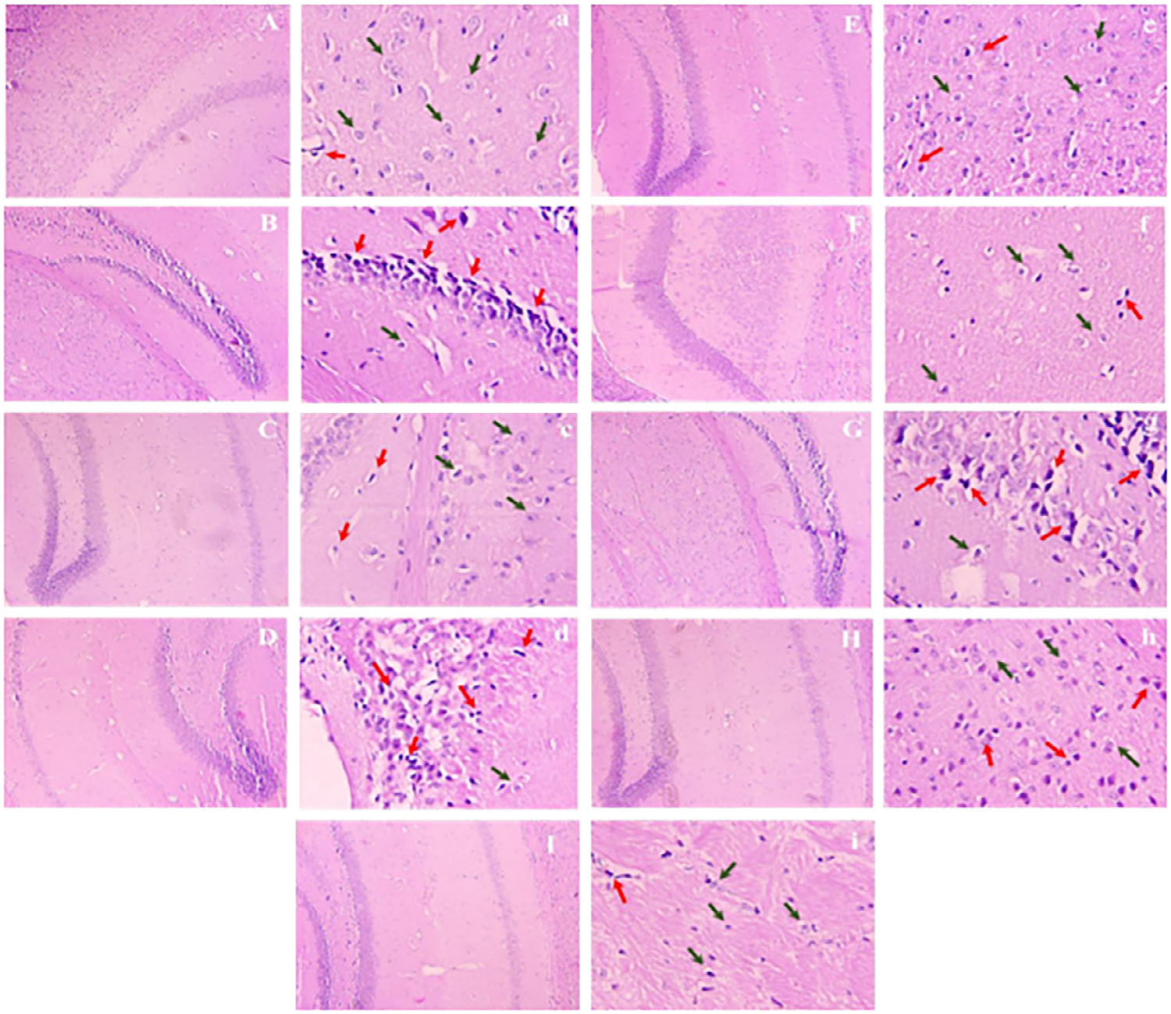# Favorable cellular alterations in cortex and hippocampal regions of mice brain by Quercetin & Naringenin: Promising role in insulin resistance induced Alzheimer’s disease

**DOI:** 10.1002/alz.087628

**Published:** 2025-01-09

**Authors:** Sukhmanpreet Kaur, Anil Kumar

**Affiliations:** ^1^ UIPS, CHANDIGARH India; ^2^ UIPS, CHANDIGARH, Punjab India

## Abstract

**Background:**

Alzheimer’s disease is a brain disorder that causes neurodegeneration and is linked with insulin resistance at molecular, clinical, and demographic levels. Defective insulin signaling promotes Aβ aggregation and accelerates Aβ formation in the brain leading to Type III diabetes.

**Objective:**

The objective of this research project is to demonstrate a linkage if any between the risk of developing Alzheimer’s disease and insulin resistance. This linkage would be addressed with Quercetin & Naringenin as a test substance using the behavioral model of Alzheimer’s disease.

**Method:**

Diabetes was induced in mice by injecting Alloxan (100mg/kg i.p.) & orally administering sucrose 35% solution. Behavioral models employed in the present study were the Morris water maze (MWM), actophotometer, and novel object recognition (NOR) test. Biochemical estimations were carried out by measuring mitochondrial, glutathione, catalase, SOD, lipid peroxidase activity, and acetylcholinesterase activity in the brain tissue. Histopathological evaluation of the hippocampal region was carried out under a bright field microscope.

**Result:**

Alloxan (100mg/kg i.p.) & sucrose 35% solution (p.o.) administration produced significant hyperglycemia in mice as compared to the control group. Alloxan administration did not produce any significant effect on weight change and locomotor activity. During the acquisition test (learning), alloxan‐treated mice significantly enhanced the escape latency time taken to reach the submerged platform. However, Naringenin administration decreased the escape latency time indicating a significant improvement in memory performance. In the Novel Object Recognition test, alloxan‐treated mice spent more time near the familiar object, and the Naringenin‐treated group spent more time near the novel object. Alloxan treatment also disrupted the mitochondrial levels and lowered the catalase, glutathione, and SOD levels, and increased the lipid peroxidase and acetylcholinesterase activities. Whereas the Quercetin & Naringenin‐treated animals restored these pathological alterations.

**Conclusion:**

Administration of Quercetin & Naringenin significantly reversed alloxan‐induced behavioral, biochemical, and cellular alterations in both the frontal cortex as well as in hippocampus. These findings suggest that quercetin may be looked upon as a promising memory‐enhancing agent in preclinical studies. Therefore, clinical trials may be undertaken to establish the memory‐enhancing property of quercetin.